# A Hybrid LSSVR/HMM-Based Prognostic Approach

**DOI:** 10.3390/s130505542

**Published:** 2013-04-26

**Authors:** Zhijuan Liu, Qing Li, Xianhui Liu, Chundi Mu

**Affiliations:** 1 Department of Automation, Tsinghua University, Beijing 100084, China; E-Mails: liqing@tsinghua.edu.cn (Q.L.); muchd@mail.tsinghua.edu.cn (C.M.); 2 Aerospace System Engineering Shanghai, Shanghai 201109, China; 3 CAD Research Center, Tongji University, Shanghai 200092, China; E-Mail: liu_xian_hui@163.com

**Keywords:** prognostics, least squares support vector regression, hidden Markov model, remaining useful life

## Abstract

In a health management system, prognostics, which is an engineering discipline that predicts a system's future health, is an important aspect yet there is currently limited research in this field. In this paper, a hybrid approach for prognostics is proposed. The approach combines the least squares support vector regression (LSSVR) with the hidden Markov model (HMM). Features extracted from sensor signals are used to train HMMs, which represent different health levels. A LSSVR algorithm is used to predict the feature trends. The LSSVR training and prediction algorithms are modified by adding new data and deleting old data and the probabilities of the predicted features for each HMM are calculated based on forward or backward algorithms. Based on these probabilities, one can determine a system's future health state and estimate the remaining useful life (RUL). To evaluate the proposed approach, a test was carried out using bearing vibration signals. Simulation results show that the LSSVR/HMM approach can forecast faults long before they occur and can predict the RUL. Therefore, the LSSVR/HMM approach is very promising in the field of prognostics.

## 1. Introduction

Condition-based maintenance (CBM), which can increase system maintenance efficiency and reduce life cycle cost, is gaining popularity. Developing the capability of prognostics and remaining useful life (RUL) prediction can save great costs and improve the logistics support. Therefore, prognostics and RUL prediction can improve the functions of CBM [1,2].

At present, various health management systems are being gradually proposed and applied, such as health and usage monitoring system (HUMS) [3], integrated vehicle health management (IVHM) [4], and prognostics and health management (PHM) [5,6]. PHM, proposed in Joint Strike Fighter (JSF), represents the highest level of CBM. In these health management systems, prognostics is an important but difficult task. Currently, there is limited research in this field due to the fact that small fault symptoms and disturbances cause difficulties in carrying out studies. Therefore, this paper aims to contribute to the growing body of research in the area of prognostics technology.

Prognostic approaches can be placed into three categories: physical model-based prognostics, evolutionary or trending model-based prognostics, and experience-based prognostics [7]. The physical model-based method has the highest accuracy, but it is often complicated to model precisely and the cost is high. Evolutionary or trending-based prognostics are data-driven and feature-based methods. They are considered effective and available prognostics approaches. The third category, experience-based prognostics, relies on statistical reliability. Some popular prognostic methods of the second category are the auto-regressive moving average (ARMA) model [8], the artificial neural network (ANN) [9], the Bayesian network [10], and the support vector regression (SVR) [11,12]. Among these methods, ANN is a widely-used method. The disadvantages of ANN are that the number of hidden layers is difficult to choose and that the calculation may fall into a local minimum. SVR is a machine-learning algorithm based on Vapnik-Chervonenkis (VC) theory and the structural risk minimization principle; therefore it avoids the disadvantages of ANN [11] and is a potential method in prognostics. There are also other extended SVR methods, such as least squares support vector regression (LSSVR) [13,14]. LSSVR proposed by Suykens [15,16] is often applied to predict non-linear time-series signals. In health management, LSSVR can be used to estimate the future health deterioration and can be extended for remaining useful life (RUL) prediction. There are a series of articles about the use of LSSVR in prognosis and monitoring, such as [17,18]. Although the LSSVR algorithm can predict the signal trends, it is difficult to assess the health status of a system for each stage. To solve this problem and make health assessments more clear, in this paper a hidden Markov model (HMM) method is introduced to describe health states during component degradation. The HMM technique, initially introduced by Baum [19], is a stochastic approach based on Markov chains. HMM has strong fault classification and status classification ability, therefore, the HMM method can be used in fault detection and diagnosis [20–22], but it cannot predict the future health status.

In this paper, a hybrid fault prognosis method based on LSSVR and HMM is proposed. In this method, LSSVR is used to predict the fault features, while HMMs are used to describe health states. Fault processes can be divided into several stages and HMMs are built and trained for each stage. Suppose given an observation sequence, the probabilities of the observation for HMMs are used to determine which health state of the given observations the sequence belongs to. Combining these HMMs with the future features predicted by LSSVR can provide an estimate of the remaining useful life (RUL) of a system.

The remainder of the paper is organized as follows: Section 2 outlines the framework of the LSSVR/HMM prognostics approach. Section 3 presents the training and estimation algorithms of HMM. Section 4 introduces the prediction algorithm based on LSSVR. Section 5 provides an application and simulation results. Section 6 presents our conclusions.

## 2. LSSVR/HMM Based Prognostics

### 2.1. The Hidden Markov Model

**Figure 1.**
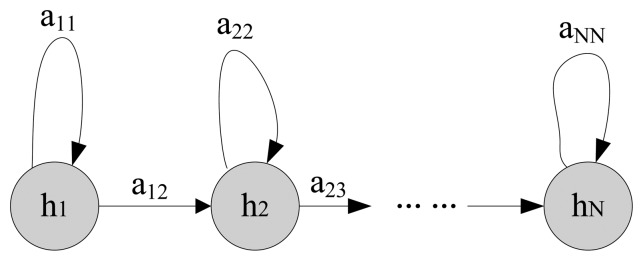
N-state left-to-right hidden Markov model.

This aim of this section is to introduce how to use the HMM-based method to describe a fault process. The fault process of a machine has a certain time-span. To analyze the fault process, it can be divided into several health states. However, fault states are unobservable but hidden in some observable signals, such as vibration signals. The HMM-based method provides a way to detect the unobservable fault states, as HMM is a stochastic technique for classifying signals and modeling [23,24]. An HMM describes a double stochastic process. One is the Markov chain, which describes state transitions. Another is the stochastic process, which describes the relations between states and observations. Suppose the number of health states is *N*, which are h_1_, h_2_,…, h*_N_*. The last state h*_N_* represents that the system fails. This state sequence is a Markov chain. The structure of an *N*-state left-to-right HMM is commonly used in fault analysis as shown in Figure 1.

As shown in Figure 1, an HMM consists of a finite number of states where a*_ij_* (*i*,*j* = 1,2,…,*N*) is the transition probability from state h*_i_* to state h*_j_*. Observation sequences from different conditions can be used to train different HMMs. For instance, observations from normal condition can train an HMM for normal state. An HMM can be described as:

(1) λ=(N,M,π,A,B)

where *N* is the number of states, *M* is the number of observations for each state, *π* is the initial probability vector, *A* = (*a_jj_*)_*N* × *N*_ is the probability transition matrix which describes the transition relations between the states, *B* = (*b_jk_*)_*N* × *M*_ describes the observation probability distribution and *b_jk_* is the probability of the *k*th observation in state *j*. *N* and *M* are known based on history experiences or prior knowledge. In other words, they are assumptions before HMM training. *π*, *A*, *B* are learned from the data. As *N* and *M* are also included in A and B, the model can be abbreviated as [23]:

(2) λ=(π,A,B)

As the observation sequence from a health state can train an HMM, N HMMs can be trained, which are written as *λ*_1_, *λ*_2_,…, *λ_N_*. *λ_i_* represents the HMM trained based on the given observation sequence from the ith health state. Given an observation sequence *O*={*o*_1_, *o*_2_,…, *o_T_*} where T is the length of the sequence, *P*(*O*|*λ_i_*) is the likelihood probability of observation sequence *O* occurring at the condition of model *λ_j_*, which can be used to determine whether *O* belongs to the health state h*_i_*.

As mentioned above, using HMMs can describe machine fault processes. According to the likelihood probabilities of present observations, HMMs can detect and diagnose faults. However, the HMM method cannot predict future health states and the RUL, because the future observation sequence cannot be obtained. To solve this problem, a hybrid prognostics method based on LSSVR and HMM is proposed in this paper, where LSSVR provides a way to predict the future fault features.

### 2.2. Framework of LSSVR/HMM Prognostics

**Figure 2.**
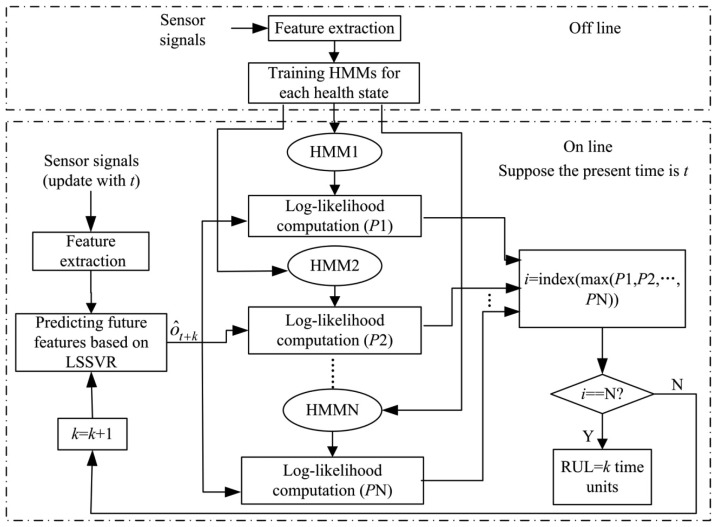
Framework of the LSSVR/HMM fault prognostics scheme which contains two processes. One is HMMs training (off line) and another is fault feature and RUL prediction process (on line).

The framework of the hybrid fault prognosis scheme is described in Figure 2. In this scheme, the LSSVR algorithm is used to predict the future fault features and the HMMs are used to describe different health states.

**Figure 3.**
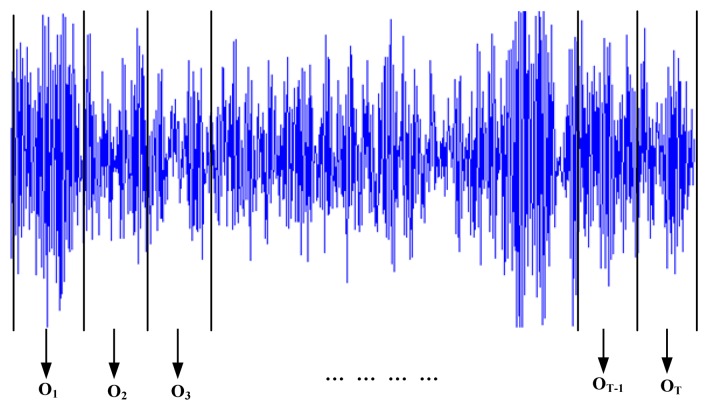
Feature extraction by segmenting the vibration signals into small windows.

As shown in Figure 2, the process of the hybrid LSSVR/HMM prognostic approach can be summarized in the following steps:

(1) Feature extraction
  As most observation signals have much random noise and uncertain interferences, features should be extracted from the signals before fault diagnosis and prognosis. In this paper, the linear predictive (LP) method, as mentioned in article [25], is used to extract the features from vibration signals. In the LP method, the signal *S_n_* can be predicted as a linear combination of the *ρ* previous signal, which can be written as:
  (3) $${\hat{s}}_{n}=l_{1}s_{n-1}+l_{2}s_{n-2}+\cdots +l_{\rho }s_{n-\rho }=\sum_{i=1}^{\rho }l_{i}s_{n-i}$$
  where *l*_1_, *l*_2_, …, *l_ρ_* are linear prediction coefficients which can be used as feature vector of the signals.
  The residual errors are written as:
  (4) $$e_{n}=s_{n}-{\hat{s}}_{n}=s_{n}-\sum_{i=1}^{\rho }l_{i}s_{n-i}$$
  The value of the linear prediction coefficients *l*_1_, *l*_2_, …, *l_ρ_* can be calculated by minimizing the mean-square value of the residual errors over a signal window. The mean square value is written as:
  (5) $${\tilde{e}}_{n}=\sqrt{\sum_{m}e_{n}^{2}(m)}=\sqrt{\sum_{m}{\left|s_{n}(m)-\sum_{i=1}^{\rho }l_{i}s_{n-i}(m)\right|}^{2}}$$
  where *e_n_*(*m*) = *e* (*n* + *m*), *S_n_*(*m*) = *S*(*n* + *m*), *n* is the starting point of the signal window, *n* + *m* is the terminal point of the signal window.
  Vibration signals are always non-stationary. If signals are segmented into several short-time signals, they can be considered stationary. Therefore, we can obtain short-time signals by segmenting vibration signals into small windows; and each window as a frame. To segment the vibration signals into small windows, the length of a small window should be determined. Each window has equal length. Based on the given length, the vibration signals can be divided into several small windows. And then the linear prediction coefficients can be extracted from these windows. The feature extraction is described in Figure 3.
  Each window is coded into a feature vector which is written as:
  (6) $$\begin{array}{cc} o_{t}={\left[l_{t1},l_{t2},\cdots l_{t\rho }\right]}^{\text{T}}, & 1\leq t\leq T \end{array}$$
  where *o_t_* is the feature vector of the *i*th signal window, *l_t_*_1_, *l_t_*_2_, …, *l_tρ_* are the linear prediction coefficients of the *t*th signal window, *T* is the number of the windows. Then the observation sequence *O*={*o*_1_, *o*_2_, …, *o_T_*} is composed of these feature vectors, which will be used for HMM training or LSSVR prediction.
(2) HMMs training
  HMMs training can now be carried out based on the fault features history. In this paper, the historic fault features are experimental data. The training data used for a specific state come from the corresponding experimental conditions. The essence of this step is to estimate the HMM parameters for each state. Based on observation sequences from *N* different conditions, optimal HMMs *λ*_1_, *λ*_2_, …, *λ_N_* are trained for corresponding health state. This step is an off-line process.
(3) Prediction of features based on LSSVR
  Suppose the present time is *t*, and the feature vector after *k* time units is denoted *ô*_*t*+*k*_ which can be predicted based on the data before time *t* using the LSSVR algorithm. Based on the feature vector *ô*_*t*+*k*_, one can predict the system health state at the future time *t* + *k*.
(4) Log-likelihood calculation
  Calculating likelihood probabilities *P*(*ô*_*t*+*k*_ |*λ_i_*) according to forward or backward algorithms [26,27] and comparing all of these probabilities, one can find the maximum probability:
  (7) $$i=\text{index}(\max(P_{1},P_{2},\ldots ,P_{N}))$$
  where *P*_1_, *P*_2_, …, *P_N_* are the likelihood probabilities for the corresponding HMMs.
  The corresponding state h*_i_* is considered the health state of the future time *t* + *k*. Therefore, this method can be used to predict future health states. If *k* = 0, the health state is the present state.
(5) RUL prediction
  As shown in Figure 2, the fault prognosis should run until the probability for the last state model is higher than for all other state models. Then the probability *P*(*ô*_*t*+*k*_|*λ_N_*) for the last state indicates that the system will fail at the future time *t* + *k*. Therefore, the RUL is *k* time units.

## 3. HMM Training

HMM training is to obtain the optimal model and the training process needs to estimate the model parameters. An HMM is described in Equation (2). When the observations are discrete, they can be represented by distributed probabilities. However, the observations are always continuous in an actual system. In this situation, a mixture of several Gaussian distributions is used to describe observation probabilities which are written as [26]:

(8) $$b_{j}(o_{t})=\sum_{l=1}^{Q}c_{jl}N(o_{t}|\mu_{jl},\Sigma_{jl})$$

(9) $$N(o_{t}|\mu_{jl},\Sigma_{jl})=\frac{1}{{\left(2\pi \right)}^{d/2}{\left|\Sigma_{jl}\right|}^{1/2}}e^{\frac{1}{2}{\left(x-\mu_{jl}\right)}^{\text{T}}{\Sigma_{jl}}^{-1}(x-\mu_{jl})}$$

where *b_j_*(*o_t_*) is the probability of observation *o_t_* at the condition of state h*_j_*, *o_t_* is the feature vector of the *t*th window, *Q* is the number of the mixture Gaussian components, *c_jl_* is the weight of the *l*th mixture component in state h*_j_*, and *μ_jl_* and *Σ_jl_* are the mean and covariance of the Gaussian density.

Then an HMM is re-written as:

(10) $$\lambda =(\pi ,A,\mu_{jl},\Sigma_{jl},c_{jl})$$

### 3.1. Forward-Backward Algorithm

Given an observation sequence *O*={*o*_1_, *o*_2_,… , *o_T_*} and a model λ, how to calculate the probability *P*(*O*|*λ*) is a basic and important problem. Both forward and backward algorithms can solve this problem.

In a forward algorithm, define the forward variables as *η_i_*(*t*) = *P*(*o*_1_, *o*_2_, …, *o_t_*, *q_t_* = h*_i_* |*λ*),1 ≤ *t* ≤ T, which is the probability of generating *o*_1_, *o*_2_, …, *o_t_* and ending in state h*_i_*. *q_t_* represents the state attime *t*.

The recursive estimation of the forward is as follows [26,27]:

(a) Initialization:
  (11) $$\eta_{i}(1)=\pi_{i}b_{i}(o_{1})$$
(b) Forward recursion (For *t*=1,2,…,*T*−1, 1 ≤ *j* ≤ *N*):
  (12) $$\eta_{j}(t+1)=\left[\sum_{i=1}^{N}\eta_{i}(t)a_{ij}\right]b_{j}(o_{t+1})$$
(c) End:
  (13) $$P(O|\lambda )=\sum_{i=1}^{N}\eta_{i}(T)$$

In a backward algorithm, we define the backward variables as *β_i_*(*t*) = *P*(*o_t_*_+1_, *o_t_*_+2_,…, *o_T_*|*q_t_* = h*_i_*, *λ*), 1 ≤ *t* ≤ T, which is the probability of generating *o_t_*_+1_, *o_t_*_+2_,…, *o_T_* and ending in state h*_i_*.

The recursive estimation of the backward is as follows [26,27]:

(a) Initialization (1 ≤ *i* ≤ *N*):
  (14) $$\beta_{T}(i)=1$$
(b) Backward recursion (For *t* = *T*-1,*T*-2, …, 1, 1 ≤ *i* ≤ *N*):
  (15) $$\beta_{t}(i)=\sum_{j=1}^{N}a_{ij}b_{j}(o_{t+1})\beta_{t+1}(j)$$
(c) End:
  (16) $$P(O|\lambda )=\sum_{i=1}^{N}\beta_{1}(i)$$

### 3.2. Re-Estimation of the HMM Parameters

The goal of HMM training is to find the model $\lambda^{*}=\underset{\lambda }{\text{argmax}}\;P(O|\lambda )$. That means the parameters of each HMM should be re-estimated until the convergence error achieves requirements. The Baum-Welch algorithm [26] provides a solution to this problem.

Given an observation sequence ***O***, define *γ_t_*(*i*) = *P*(*q_t_* = h*_i_* |*O*, λ), which is the probability of that the state at time *t* is h*_i_*. *γ_t_*(*i*) can be calculated as:

(17) $$\gamma_{t}(i)=\frac{\eta_{i}(t)\beta_{t}(i)}{\sum_{j=1}^{N}\alpha_{j}(t)\beta_{t}(j)}$$

Define *γ_t_*(*i, l*) as the probability of the lth mixture Gaussian component when the state at time *t* is h*_i_*. *γ_t_*(*i, l*) can be calculated as:

(18) $$\gamma_{t}(i,l)=\gamma_{t}(i)\frac{c_{il}N(o_{t}|\mu_{il},\Sigma_{il})}{\sum_{m=1}^{Q}c_{im}N(o_{t}|\mu_{im},\Sigma_{im})}$$

*ξ_t_*(*i, j*) = *P*(*q_t_* = h*_i_*, *q*_*t*+*1*_ = h*_j_* |*O*, *λ*) is defined as the probability of that the state at time *t* is h*_i_* and the state at time *t* + 1 is h*_j_*. *ξ_t_*(*i, j*) can be calculated as:

(19) $$\xi_{t}(i,j)=\alpha_{t}(i)\eta_{ij}b_{j}(o_{t+1})\beta_{t+1}(j)/P(O|\lambda )$$

If there are K observation sequences and the length of each sequence is *T*, the parameters of a HMM can be estimated as follows [26,27]:

(20) $$\pi_{i}=\frac{\sum_{k=1}^{K}\gamma_{1}^{k}(i)}{K}$$

(21) $$a_{ij}=\frac{\sum_{k=1}^{K}\sum_{t=1}^{T}\xi_{t}^{k}(i,j)}{\sum_{k=1}^{K}\sum_{t=1}^{T}\gamma_{t}^{k}(i)}$$

(22) $$c_{il}=\frac{\sum_{k=1}^{K}\sum_{t=1}^{T}\gamma_{t}^{k}(i,l)}{\sum_{k=1}^{K}\sum_{t=1}^{T}\gamma_{t}^{k}(i)}$$

(23) $$\mu_{il}=\frac{\sum_{k=1}^{K}\sum_{t=1}^{T}\gamma_{t}^{k}(i,l)o_{t}^{k}}{\sum_{k=1}^{K}\sum_{t=1}^{T}\gamma_{t}^{k}(i)}$$

(24) $$\Sigma_{il}=\frac{\sum_{k=1}^{K}\sum_{t=1}^{T}\gamma_{t}^{k}(i,l)(o_{t}^{k}-\mu_{il}){\left(o_{t}^{k}-\mu_{il}\right)}^{\text{T}}}{\sum_{k=1}^{K}\sum_{t=1}^{T}\gamma_{t}^{k}(i)}$$

According to Equations (20)–(24), the parameters of the HMMs are re-estimated. Finally, optimal models of the health states can be obtained as shown in Figure 2. Given an observation, the likelihood probabilities for each state can be calculated according to Equations (13) or (16). In the forward-backward algorithm, *η_i_*(*t*) and *β_t_*(*i*) will tend to be zero as time grows. To avoid this problem, logarithmic values of probabilities are usually used in the actual calculations.

## 4. Feature Trend Prediction Based on LSSVR

### 4.1. LSSVR Algorithm

The idea of SVR is to map non-linear problems to linear problems in a high dimension space. LSSVR is an improved algorithm based on SVR, and it uses a least square loss function instead of the *ε*-insensitive loss function [16,28]. Compared with SVR, the calculation procedure of LSSVR has higher efficiency.

Considering the sample data as {(*x*_1_,*y*_1_), …, (*x_i_*,*y_i_*), …, (*x_D_*,*y_D_*)}, where ***x**_i_* is the input vector, and ***y**_i_* is the objective value. The optimization equation of the LSSVR is described as [13,14]:

(25) $$\begin{array}{l} \underset{\omega ,\kappa ,e}{\min}J(\omega ,e)=\frac{1}{2}{\left\|\omega \right\|}^{2}+\frac{1}{2}C\sum_{i=1}^{D}e_{i}^{2}\\ s.t.\;y_{i}=\omega^{\text{T}}\phi (x_{i})+\kappa +e_{i}\;1\leq i\leq D \end{array}$$

where ***ω*** is weight vector, *e_i_* is error variable, *C* is a regularization parameter used to control the punishment degree of samples beyond the error, the function of *Φ*(*) is to allow LSSVR to work in a linear space, and κ is the bias term which is an unknown and needs to be estimated.

The Lagrangian is written as:

(26) $$L(\omega ,e,\kappa ,\alpha )=J(\omega ,e)-\sum_{i=1}^{D}\alpha_{i}(\omega^{\text{T}}\phi (x_{i})+\kappa +e_{i}-y_{i})$$

where *α_i_* is Lagrange multipliers.

According to Karush-Kuhn-Tucker (KKT) optimization conditions, the optimality conditions are given as [13,14]:

(27) $$\left\{ \begin{array}{l} \frac{\partial L}{\partial \omega }=\omega -\sum_{i=1}^{D}\alpha_{i}\phi (x_{i})=0\\ \frac{\partial L}{\partial \kappa }=-\sum_{i=1}^{D}\alpha_{i}=0\\ \frac{\partial L}{\partial e_{i}}=Ce_{i}-\alpha_{i}=0\\ \frac{\partial L}{\partial \alpha_{i}}=-(\omega^{\text{T}}\phi (x_{i})+\kappa +e_{i}-y_{i})=0 \end{array}\right.$$

By solving Equation (27), the parameters *α_i_* and κ can be obtained. Then the LSSVR model for estimation is written as:

(28) $$y(x)=\sum_{i=1}^{D}\alpha_{i}K(x_{i}\cdot x)+\kappa$$

where *K*(*x_i_*·*x*) is the kernel function which satisfies the Mercer condition:

(29) $$K(x_{i}\cdot x_{k})=\phi {\left(x_{i}\right)}^{\text{T}}\phi (x_{k})$$

Frequently used kernel functions are the polynomial, sigmoid and radial basis kernel (RBF) functions. This paper applies RBF because it can classify multi-dimensional data. The RBF kernel function is given as:

(30) $$K(x\cdot x_{k})=\exp(-\frac{{\left\|x-x_{k}\right\|}^{2}}{\sigma^{2}})$$

In fault prognosis, time series are the extracted feature data. Suppose the front *r* data have been obtained, then the object is to predict the future data using the front *r* data. The input vector and the output value can be constructed as:

$$X=\left[\begin{array}{cccc} x_{1} & x_{2} & \cdots & x_{p}\\ x_{2} & x_{3} & \cdots & x_{p+1}\\ \vdots & \vdots & \vdots & \vdots \\ x_{r-p} & x_{r-p+1} & \cdots & x_{r-1} \end{array}\right],\;Y=\left[\begin{array}{c} x_{p+1}\\ x_{p+2}\\ \vdots \\ x_{r} \end{array}\right]$$

where *p* is the number of embedded dimensions. According to above LSSVR method, training this sample data can estimate the parameters *α_i_* and *κ*. Then the prediction function can be written as:

(31) $${\hat{x}}_{r+k}=\sum_{i=1}^{D}\alpha_{i}K(x_{i}\cdot \tilde{x})+\kappa$$

where *k* is the prediction steps, ***x****_i_* is the *i*th support vector, and *x̃* is *x̃* = {x_r+k-&upsilon;,_ …., *x̃_r_*_+1_,…, *x̃_r_*_+k-1_}.

As mentioned above, LSSVR can predict future fault features. However, it is difficult to describe and assess health states. Combining LSSVR with HMM, it can realize health state prediction and RUL prediction. The feature vectors predicted based on the LSSVR are used as observations in each HMM to calculate the log-likelihood probabilities as shown in Equation (13) or Equation (16). The model with the highest likelihood probability represents the system health state at the corresponding future time.

### 4.2. Algorithm Improvement

As analyzed above, two parameters *C* and *σ*in the LSSVR algorithm need to be optimized. Selecting superior parameters can improve the training speed and the performance of LSSVR. Genetic algorithms (GA) [13] and particle swarm algorithms [14] are two optimization methods used to determine the LSSVR parameters. In this paper, particle swarm optimization (PSO) is used. PSO realizes optimization by searching the partial and global optimal solution in a particle swarm. Define the fitness function of the *i*th particle as:

(32) $$J_{i}(C,\sigma^{2})=\frac{1}{\sum_{k=1}^{N}{\left(y_{k}-f(x_{k})\right)}^{2}+eps}$$

where *eps* is a small value to avoid the denominator to be zero.

**Figure 4.**
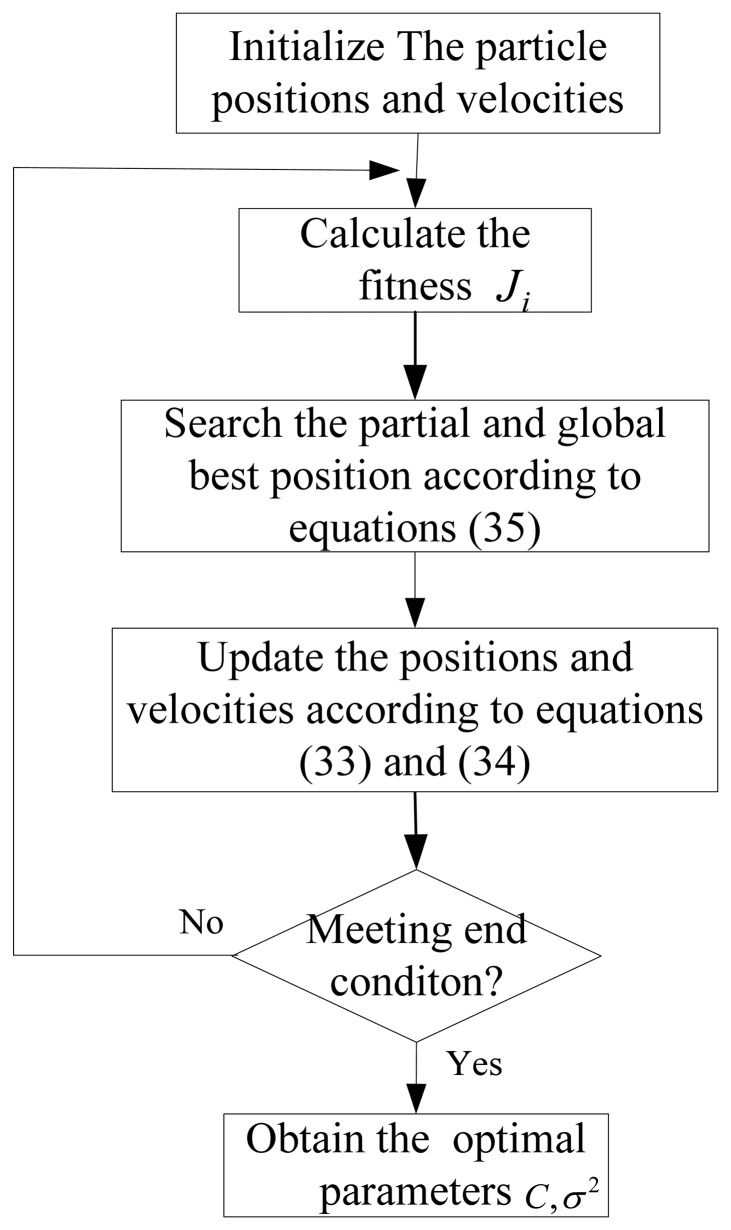
The parameters optimization process of the LSSVR based the PSO algorithm.

The particle positions and velocities are written as *X_i_*=(*x_i1_, x_i2_*,…, *x_iD_*)^T^ and *V_i_*=(*v_i1_, v_i2_*,…, *v_iD_*)^T^ (*i*=1,2, …, *m*). *m* is the number of the particles. The partial best position is written as *P_i_* and the global best position is written as *P*_g_. Then the velocity and position updating can be written as:

(33) $$v_{id}(k+1)=Iv_{id}(k)+c_{1}r_{1}(k)(P_{id}(k)-x_{id}(k))+c_{2}r_{2}(k)(P_{gd}(k)-x_{id}(k))$$

(34) $$x_{id}(k+1)=x_{id}(k)+v_{id}(k+1)$$

where *v*_*id*_(*k*) and *x*_*id*_(*k*) are the *d*th dimension velocity and position of the *i*th partial in the *k*th iteration, *I* is an inertia factor, *c*_1_ and *c*_2_ are study factor, *P_id_* and *P_gd_* are the *d*th dimension partial and global best position in the *k*th iteration, *r*_1_ and *r*_2_ are random during [0,1]. The optimization process is described as Figure 4, where the initial positions and velocities are given randomly among the range of the particle swarm.

In Figure 4, the partial and global best position searching process is as follows:

(35) $$\begin{array}{llll} \text{if}\;J_{i}>J_{\text{best}}, & \text{then} & P_{i}=X_{i}, & J_{\text{best}}=J_{i}\\ \text{if}\;J_{i}>G_{\text{best}}, & \text{then} & P_{g}=X_{i}, & G_{\text{best}}=J_{i} \end{array}$$

where *J*_best_ is the partial best fitness and *G*_best_ is the global best fitness.

The end condition is meeting the accuracy requirements or arriving at the maximum iterations. When meeting the end condition, the best position is considered as the optimal parameters used in LSSVR.

As time goes by, new observations are added which may include fault information and can improve prediction accuracy. However, the training data will become large with time growth. If the number of sample data is too large, it will affect training efficiency. To solve this problem, some unimportant old data, such as non-support vector samples or far history samples, are selected for deletion in this paper. These data will not affect the training accuracy seriously. By adding new data and deleting some old data, the training samples sequence will be renewed online with time growth. The new training sample can be written as:

(36) $$X(t+1)=X(t)+X_{\text{new}}(t+1)-X_{\text{del}}(t)$$

where *X*(*t*) is the training sample at time *t*, *X*_new_(*t*+1) is the added new sample at time *t* + 1, *X*_del_(*t*) is the deleted sample chosen from *X*(*t*).

**Figure 5.**
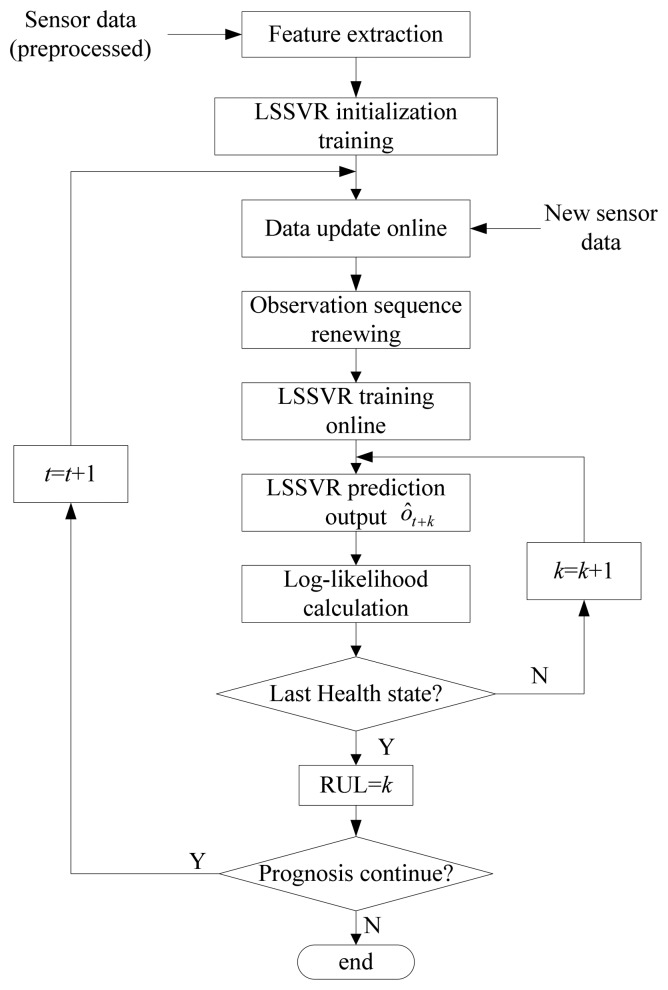
RUL prediction process based on the improved LSSVR with data renewed online.

The RUL prediction process based on the improved LSSVR algorithm is summarized in Figure 5.

As shown in Figure 5, at each time *t*, RUL equals to the future prediction time whose corresponding state is the last health state. The prediction accuracy of RUL increases as time increases because new observations are added and some unimportant old data are deleted, which makes the feature trend predictions more accurate.

## 5. Simulations and Results

To evaluate the performance of the LSSVR/HMM prognostic approach, it was tested using experimental bearing vibration data from the Case Western Reserve University Bearing Data Center [29]. The bearing test stand consists of a 2 hp motor, a torque transducer/encoder, a dynamometer, and control electronics. The test bearings support the motor shaft. The details of the test stand are shown in the website [29]. The actual test conditions of the motor as well as the bearing fault status have been carefully documented for each experiment. Different levels of faults in diameter were collected separately for the inner raceway, rolling element (*i.e.*, ball) and outer raceway. Faulted bearings were reinstalled into the test motor and vibration data was recorded.

In our simulation, the inner race vibrations signals are used. Its fault diameter includes the following levels: 0.007 inches, 0.014 inches, and 0.021 inches. The data was sampled at 12 kHz. Added to the normal states, fault process can be divided into four health states. The bearing data center provides vibration data from different health states. Data from different conditions are discrete, which can be used to train HMMs for the corresponding health status.

### 5.1. Feature Extraction

**Figure 6.**
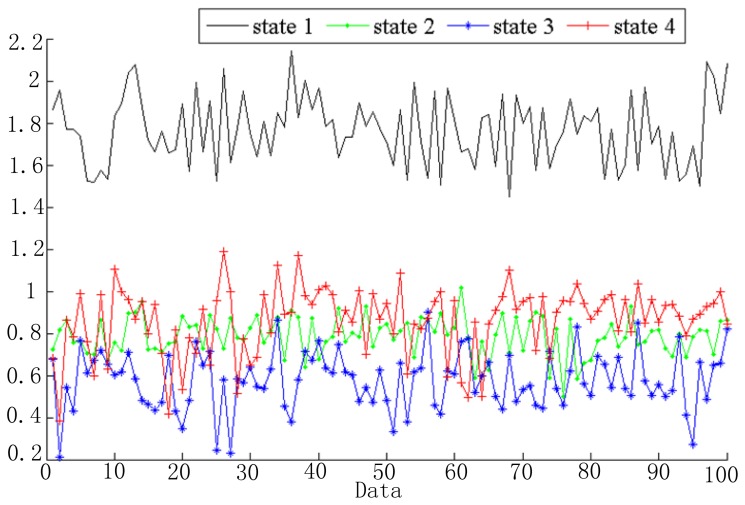
The first dimension of the feature vectors for each health states.

Vibration data are segmented into several windows and extracted feature vectors as mentioned in Section 2.2 and Figure 3. In the simulation, 256 sample data were chosen as a frame. Based on the linear predictive method, signals in each window can be predicted as a linear combination of its *ρ* previous signal, which is shown in Equation (3). In the simulation, we take *ρ* as 8. Then the feature vector of each signal window is *o_t_* = [*l_t_*_1_, *l_t_*_2_, …, *l_t_*_8_]^T^ and the number of the feature dimensions is 8. Take the first dimension as an example; the features of the four health states are shown in Figure 6. As shown in Figure 6, the extracted feature for each health state is different. This demonstrates that these features can be used to distinguish different fault states.

### 5.2. HMMs Training and Fault Diagnosis

Suppose three Gaussian distributions are used to describe observation probabilities for each state. Give the initial HMM parameters as follows:

$$\begin{array}{c} \pi ={\left[1,0,0,0\right]}^{\text{T}}\\ A=\left[\begin{array}{cccc} 0.5 & 0.5 & 0 & 0\\ 0 & 0.5 & 0.5 & 0\\ 0 & 0 & 0.5 & 0.5\\ 0 & 0 & 0 & 1 \end{array}\right] \end{array}$$

Generally speaking, the influence of the initial values of *π* and *A* is not large. But the initial values of *μ_jl_*, *U_jl_*, and *c_jl_* may affect the training result. They can be calculated using K-means method based on the given samples, which are not discussed here in detail. The convergence error is given as 0.00001.

**Figure 7.**
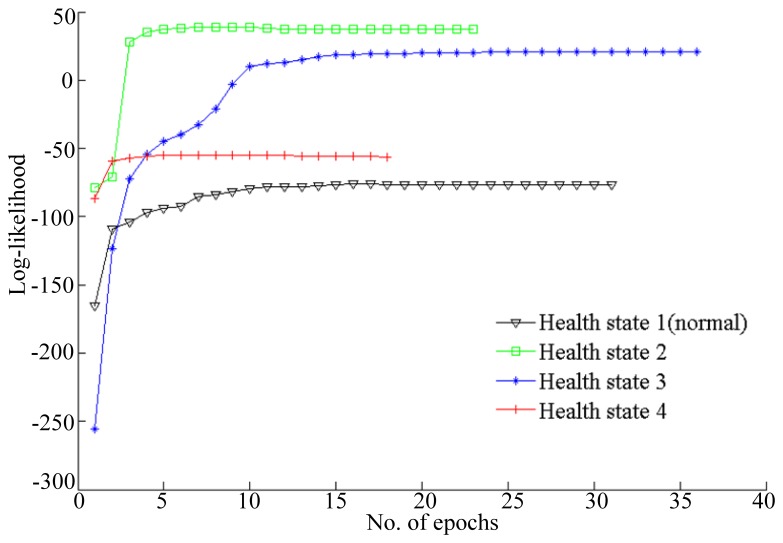
Log-likelihoods of HMMs during training for the four states.

To improve the training accuracy, several observation sequences are used to train HMM for a health state. The log-likelihood probabilities of HMMs training are shown in Figure 7.

**Figure 8.**
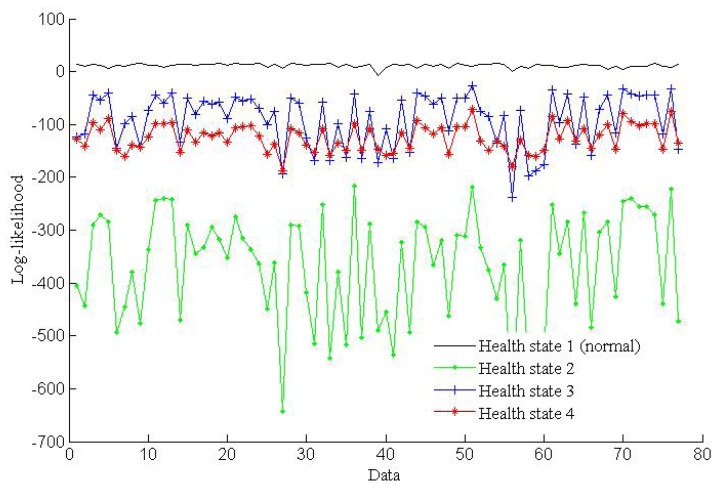
Log-likelihoods for the four state HMMs of the fault-free test data.

**Figure 9.**
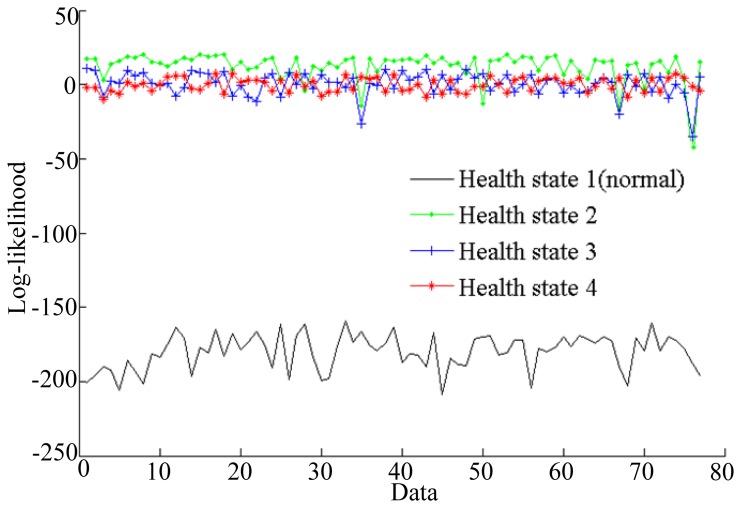
Log-likelihoods for the four state HMMs of the test data.

The simulation results shown in Figure 7 illustrate that the log-likelihoods tend to stabilize with the growth of the number of epochs. All of the training epochs are less than 40. These trained HMMs can be used to diagnose faults. In this paper, two vibration data sequences are specimens for fault diagnosis testing, where one is fault-free data and another is data with a fault diameter of 0.007 inches. The log-likelihood probabilities of the test vibration data with fault-free are shown in Figure 8, while the log-likelihood probabilities of the data with fault diameter of 0.007 inches are shown in Figure 9.

As shown in Figure 8, the HMM with the highest probability is health state 1, which represents fault-free state. That is to say the test data are fault-free. The result is consistent with the given data. Figure 9 shows that the HMM with the highest probability is health state 2, which represents the fault diameter of 0.007 inches. Therefore, the diagnosis result is consistent with the given data. According to the above two tests, fault diagnosis based on HMMs is efficient.

### 5.3. Features and RUL Prediction

**Figure 10.**
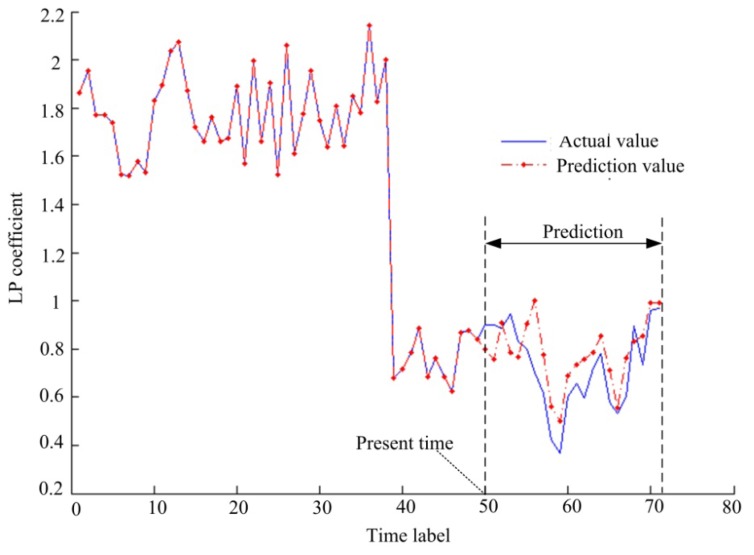
The prediction result of the first dimension of the feature vectors based on the LSSVR.

Collecting lifetime data is a difficult work and the bearing data center does not provide this lifetime data. Therefore, we construct a lif time data sequence by taking some vibration data separately from each health states. Then the constructed lifetime data sequence is used to test for features prediction and fault prognostics. After frame segmentation and feature extraction, the length of the feature sequence is 71. We take the front 50 feature vectors as LSSVR training data, and the back 21 as unknown test data. With these parameters, the present time is *t* = 50 and the future prediction time is *k* = 21. We take the first dimension of the feature vectors as an example, and the prediction result is shown in Figure 10.

In Figure 10, the data before *t* = 50 are regression estimation results and the data after *t* = 50 are prediction results according to Equation (28). The simulation results indicate that the prediction feature trend is essentially consistent with the actual trend. The simulation results can therefore be used to make a fault prognosis.

**Figure 11.**
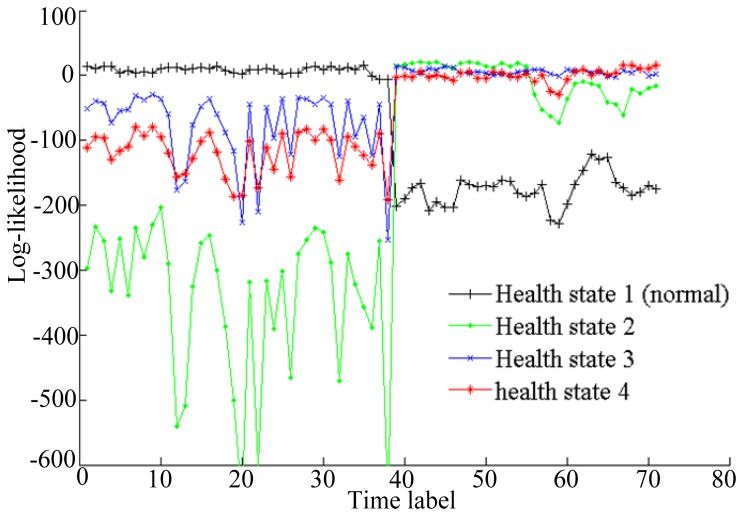
Log-likelihoods for the four state HMMs of the test life time data.

Based on the trained four HMMs, the log-likelihood probabilities of the life time data for each state are shown in Figure 11.

The state with the highest log-likelihood is the health state of the corresponding time. As shown in Figure 11, the system goes from health state 1 to health state 4 during the lifetime. The system is in a normal state before *t* = 38. During the time from *t* = 39 to *t* = 55, the system is in health state 2 which means the fault diameter is at 0.007 inches. During the time from *t* = 56 to *t* = 65, the system is in health state 3, which means the fault diameter is at 0.014 inches. After *t* = 66, the system is in health state 4. That indicates that the system fails after *t* = 66. RUL values can be predicted at each time. The RUL prediction results at different times are shown in Table 1.

In Table 1, the actual value of RUL is the time between the present time and the failure time *t* = 66. The predicted value of RUL is calculated based on the proposed LSSVR/HMM method. As shown in Table 1, the predicted RUL is less than the actual RUL. Therefore, the LSSVR/HMM hybrid prognosis method can forecast faults long before the actual fault occurs. The RUL prediction errors reduce with increasing time and it tends to be 1 time unit in the simulation, but in real situations, the prediction results may be not so good because the test lifetime data used in the simulation is perfect and shorter than an actual lifetime data sequence.

## 6. Conclusions

Fault prognosis is an essential and difficult technology in health management and CBM. In this paper, a hybrid prognostics approach which integrates the HMM method and LSSVR is presented. In the proposed LSSVR/HMM approach, the fault process is divided into several health states modeled by HMMs. These HMMs are trained based on history sample data. The lot-likelihood probabilities of the HMMs are used to classify data and determine the health state. The state with the highest log-likelihood is the corresponding health state of given data. LSSVR is used to predict the feature trend. Based on the predicted features and trained HMMs, the future health state can be predicted. LSSVR trains and predicts RULs online until the highest probability of the predicted feature vector is at the last health state. Then the RUL is equal to the prediction time.

Testing of the proposed LSSVR/HMM prognostics approach was carried out using bearing vibration data. The simulation results showed that the novel approach is efficient in fault diagnosis and prognostics and can also predict the RUL. Furthermore, the results showed that the RUL prediction accuracy increases with the passing of time. Future work will focus on how to improve the prediction accuracy and expand the applications.

## Figures and Tables

**Table 1.** Remaining useful life prediction results.

**Present time**	50	51	52	53	54	55	56	57	58	59	60
**Actual RUL**	16	15	14	13	12	11	10	9	8	7	6
**Predicted RUL**	9	10	10	11	9	10	9	8	7	6	5
**Prediction errors**	7	5	4	2	3	1	1	1	1	1	1
